# Moving from ‘fully’ to ‘appropriately’ informed consent in genomics: The PROMICE framework

**DOI:** 10.1111/bioe.13027

**Published:** 2022-04-07

**Authors:** Julian J. Koplin, Christopher Gyngell, Julian Savulescu, Danya F. Vears

**Affiliations:** ^1^ Biomedical Ethics Research Group Murdoch Children's Research Institute Melbourne Victoria Australia; ^2^ Melbourne Law School University of Melbourne Melbourne Victoria Australia; ^3^ Department of Paediatrics University of Melbourne Melbourne Victoria Australia; ^4^ Faculty of Philosophy, Oxford Uehiro Centre for Practical Ethics Oxford University Oxford UK

**Keywords:** autonomy, genomics, informed consent, rapid genomic testing

## Abstract

Genomic sequencing technologies (GS) pose novel challenges not seen in older genetic technologies, making traditional standards for fully informed consent difficult or impossible to meet. This is due to factors including the complexity of the test and the broad range of results it may identify. Meaningful informed consent is even more challenging to secure in contexts involving significant time constraints and emotional distress, such as when rapid genomic testing (RGS) is performed in neonatal intensive care units. In this article, we propose that informed consent matters not for its own sake, but because obtaining it furthers a range of morally important goals, such as promoting autonomy, well‐being, and trust in medicine. These goals form the basis of a new framework [PROmoting Morally Important Consent Ends (PROMICE)] for assessing the ethical appropriateness of various informed consent models. We illustrate this framework with two examples: (a) a tiered and layered consent model for obtaining consent for GS, and (b) consent for RGS in critically ill newborns. We conclude that *appropriately*—rather than *fully*—informed consent provides the correct standard for genomic medicine and research.

## 1 INTRODUCTION

Genomic sequencing technologies (GS) have enormous potential to improve human well‐being, both in clinical settings and via genomic research. However, GS pose novel ethical challenges not seen in standard genetic techniques. These include questions about how to manage secondary findings (information that is not explicitly sought),^1^ as well as challenges in obtaining informed consent.^2^ This article explores this latter challenge.

In medicine and research, ‘consent’ occurs when A (who might be a patient or a research participant) acquiesces to B (who might be a physician or researcher) doing something to A (such as administering a medical test). Consent is said to be *informed* when A has received relevant information and has appropriate decision‐making capacity. Consent is said to be *fully* informed when ‘a capacitated (or ‘competent’) patient or research participant to whom full disclosures have been made and who understands fully all that has been disclosed agrees voluntarily to treatment or participation on this basis’.^3^

The standard of fully informed consent can be difficult or impossible to meet in the context of GS. Genomic information is highly complex and difficult to understand. The range of possible results from genomic sequencing is often very broad, meaning it is difficult to convey the possible consequences that could follow sequencing. This challenge is exacerbated by the possibility that genomic sequencing will reveal findings unrelated to the condition being investigated (referred to as incidental or unsolicited findings) or variants of uncertain clinical significance (where a DNA change cannot be classified as either benign or pathogenic based on current evidence). The complexity of genomic information and the range of possible findings render fully informed consent unattainable; it is not feasible to ensure that patients and/or research participants understand the full range of possible outcomes of testing.^4^ Genetic health professionals—including genetic counsellors—have reported struggling to fully prepare patients for the range of results they might receive.^5^ The challenges in securing informed consent can be even greater in the face of significant time constraints and emotional distress, such as when rapid genomic testing is performed in neonatal intensive care units.^6^

Even if patients are not, in practice, required to be told about every specific condition, genomic sequencing has such an enormous array of potential consequences that it may not be practical to convey all the meaningful impacts the tests could have on people's lives. This challenge is becoming widely recognized, and a range of innovative informed consent models are being developed in response. However, what the literature lacks—and what we attempt to provide—is a moral standard against which to assess these models. Such a standard can help structure critical reflection on the various informed consent models currently under discussion in the genomics literature, as well as help drive the development of new ones. It can help us determine where departures from fully informed consent are appropriate.

We propose a new moral framework for assessing informed consent models, which is grounded in four moral goals: respecting autonomy, promoting autonomy, promoting well‐being, and preserving trust in medicine. This framework is intended to aid decision‐making about how informed consent should be operationalized in contexts where ‘fully’ informed consent is unfeasible. It does so by outlining the key factors by which different approaches to informed consent ought to be evaluated—that is, how well different approaches achieve the underlying moral goals that informed consent is meant to promote.

GS is only one domain where securing meaningfully informed consent raises practical challenges and conceptual questions.^7^ We hope our analysis will also prove useful outside of genomics. This article, however, focuses on the context of GS, a domain with which we are especially familiar, and in which the challenges for informed consent are widely recognized, acute, and more pronounced than in genetic testing using older sequencing technologies.^8^

## 2 INFORMED CONSENT'S GOALS

What are the moral goals of informed consent? Consent plays a unique role in moral and legal theory, where it is often considered morally transformative. The ‘moral magic’ of consent, as some theorists describe it, is that consent renders otherwise impermissible acts permissible. Consent is what: ‘… turns a rape into love‐making, a kidnapping into a Sunday drive, a battery into a football tackle, a theft into a gift, and a trespass into a dinner party’.^9^

However, in some contexts—including medicine and medical research—consent *by itself* is not considered sufficient to transform impermissible acts into permissible ones. Instead, the appropriate moral yardstick is *informed* consent—a benchmark that requires, inter alia, that the patient has been educated about the risks and benefits of, and alternatives to, a given intervention. The ethical importance of informed consent is widely accepted in medicine.^10^ Surprisingly, however, there is no canonical account of the underlying moral justification for informed consent requirements, nor the goals they are meant to promote.^11^

It is often treated as a ‘truism’ that informed consent is justified by the principle of respect for autonomy,^12^ to the extent that this is the primary justification for seeking informed consent given in bioethics textbooks.^13^ More recently, it has been argued that we should seek to promote, rather than merely respect, patients’ autonomy.^14^ For reasons we describe below, promoting autonomy can be seen as an independent goal from respecting autonomy, both of which can be achieved through informed consent. An alternative view expressed in the literature ties the value of informed consent, not to autonomy, but to well‐being.^15^ As people are generally in the best position to know what will make their life go best; informed consent empowers individuals to promote their own well‐being. In addition, in her book *Autonomy and trust in bioethics*, Onora O'Neill argues that an alternative justification for informed consent is to promote trust in medicine.^16^

While these four goals of informed consent (to respect personal autonomy, promote autonomy, promote well‐being, and preserve trust in medicine) have been independently argued for in the bioethics literature, it is important to note that they are not mutually exclusive. In this article we take a pluralist approach to the value of informed consent, whereby informed consent is morally important because it jointly performs four morally important functions. Each of these four functions will form a pillar of the PROmoting Morally Important Consent Ends (PROMICE) framework.

The following section further explicates each of four morally important goals that can be secured through informed consent, beginning with the most common view: that the value of informed consent requirements is that they respect patients’ autonomy.^17^

### 2.1 Respecting and promoting autonomy

Autonomy, at its most basic level, refers to self‐rule or self‐governance. It involves forming one's own values about what would make one's own life go as well as possible, or about how one should act morally. We exercise autonomy when we choose options that fit our own values and plan of life. In political philosophy, respect for autonomy is often conceptualized as a negative obligation duty to abstain from interfering with a person's actions.^18^ We respect personal autonomy when we allow people to make their own decisions; conversely, we fail to respect others’ autonomy when we seek to bring their decisions under our control. This negative obligation requires medical professionals to abstain from coercing or deceiving patients. To coerce a patient into a particular course of action, or to mislead them about the nature or risks of a particular procedure, clearly evinces a lack of respect for the patient's autonomy; coercion and deception usurp patients’ control over the direction of their own lives. Respect for autonomy requires medical professionals to ensure their patients consent to how they are treated, and to abstain from subjecting them to controlling influences.

These negative obligations do not, by themselves, offer a complete justification for informed consent requirements. This is because informed consent requirements are not merely an expression of genetic health professionals’ *negative* obligations toward patients; they also involve a separate *positive* obligation to help patients understand the risks and benefits of genomic testing. Consider a scenario in which a clinician fails to disclose risk information not because they are trying to manipulate their patient into a particular course of action, but rather because they feel they are too busy, or have simply forgotten, to secure informed consent. In this case, the clinician does not consciously seek to bring the patient under their own control; although they fail to disclose relevant information, they do not deceive, coerce, or otherwise push the patient towards a particular course of action. The clinician has clearly done something wrong—clinicians *do* have a moral obligation to secure informed consent, no matter how busy or forgetful they might be—but they have not attempted to usurp their patient's autonomy.^19^ Obtaining *consent* is necessary to respect autonomy and meet one's negative obligations (since otherwise the clinician would be coercing their patient); obtaining *informed* consent is not.^20^ Although respect for autonomy (conceived in terms of negative obligations against interference) is a crucially important value in medical ethics, it does not fully account for informed consent's moral importance.

The goal of respecting autonomy needs to be supplemented with a second one: the goal of promoting autonomy. Clinicians do not only have negative duties not to usurp patients’ autonomy, but also positive duties to *promote* autonomy.^21^ The degree to which we are autonomous depends, in part, on whether we understand the true nature of our actions and their likely consequences.^22^ The reason that information is important to autonomy is that it enables patients to accurately conceptualize the nature of options on offer, and consider these in relation to their own psychology, wants and beliefs. It enables them to fit the world to their desires accurately.^23^ A moral obligation to *promote* autonomy can neatly account for the wrongfulness of inadvertently or negligently failing to secure informed consent. This is because an obligation to promote autonomy requires us to actively try to facilitate autonomous decision‐making. This would involve, inter alia, providing patients with information that can help them determine which option best fits their own values. To fail to provide patients with sufficient relevant information (or to fail to provide information that will be understood) can make it difficult for patients to determine what course of action best accords with their values.

Admittedly, autonomous decision‐making requires more than mere understanding of the possible outcomes of our actions. For example, it might also require us to reflect on our goals and preferences.^24^ Accordingly, securing informed consent does not *by itself* ensure persons are acting autonomously. However, since *failing* to secure informed consent undermines the possibility of autonomous decision‐making, informed consent requirements can nonetheless be said to help promote autonomy.

This analysis points toward an important distinction between clinicians’ negative duties to respect autonomous decisions and positive duties to promote autonomy. This tracks an influential distinction in political philosophy between negative and positive liberty. Isaiah Berlin's ‘Two concepts of liberty’ provides an early and influential articulation of this distinction. For Berlin, negative liberty involves the absence of constraints imposed by others, whereas positive liberty involves one's actual ability to pursue and achieve one's goals.^25^ The promotion of positive and negative liberty are seen, by Berlin and others, as distinct political projects.^26^

In bioethics, positive and negative obligations are sometimes blurred together under the umbrella category of ‘respect for autonomy’. The thought here is that full respect for patients’ autonomy includes not only negative obligations against interfering with autonomous choices, but also positive obligations to foster autonomous decision‐making. As shown above, however, there are important distinctions between these goals, with positive obligations to promote autonomy providing most of the justification for informed consent requirements. We treat these negative and positive duties separately. We restrict the label ‘respect for autonomy’ to refer to negative duties against interfering with autonomous choices, and we use the term ‘promotion of autonomy’ to refer to positive duties to aid in decision‐making. Both are crucially important to informed consent.

### 2.2 Promoting (and protecting) well‐being

The value of informed consent might also consist partly in the promotion of personal well‐being,^27^ not merely the promotion of autonomy.^28^ In considering personal well‐being, we operate under the premise that what promotes one's well‐being depends, at least in part, on one's own conception of the good, or one's own value set. Hence, there is reason to think that individuals tend to be particularly well positioned to judge what will promote one's own well‐being (although this will not necessarily always be the case). In relation to informed consent, one might predict that informed patients would generally be better able to promote their own well‐being. On this view, informed consent requirements are instrumentally valuable because securing informed consent will typically promote well‐being. In this sense, the goals of promoting autonomy and promoting well‐being will generally run together.

The counterpart of a duty to promote well‐being is a duty to protect it. To use the language of Beauchamp and Childress's *Principles of biomedical ethics*,^29^ clinicians not only have duties of beneficence, but also duties of non‐maleficence. In the context of genomic testing, a lack of informed consent can cause harm. This might occur if a patient has an unrealistic idea about the probability that the test will identify the cause of their condition, or if testing identifies an unsolicited finding and they were not forewarned of this possibility. In this sense, too, informed consent helps patients choose the options that align with their interests and promote their well‐being.

However, one might question whether individuals *are* always best placed to judge what is good or bad for them, and—by extension—whether informed consent is always the most reliable path to promoting well‐being. Human reasoning is subject to a range of heuristics and biases that sometimes cause us to act contrary to our own underlying goals.^30^ It can be particularly difficult to choose the best course of action when information is complex and highly technical in nature, such as in genomics. In these contexts, doctors have expertise and experience that patients lack. Insofar as the value of informed consent is grounded (wholly or partly) in the promotion of well‐being, then, at least in principle, some degree of non‐coercive paternalism might be appropriate.^31^ Guiding patients towards a particular course of action involves taking on a broader role than merely securing informed consent—and such directiveness would, strictly speaking, conflict with the requirements of informed consent.^32^ However, insofar as concerns about informed consent amount to concerns about well‐being, directiveness might be appropriate to the extent that it would more effectively promote patients’ interests (and thereby achieve one of informed consent's underlying goals). Another way to put this is to say that insofar as informed consent requirements are merely a means to promoting well‐being, it may sometimes be appropriate to depart from these requirements when we can more effectively promote well‐being in other ways.^33^

These questions about how to make trade‐offs between informed consent and well‐being are complex, and they demand more attention than we can give them here. In many cases, however, it will not be necessary to wade into them. For the sake of this article, our key point is that informed consent usually (though not necessarily always) helps to promote well‐being and avoid harm. This function is one of the key moral purposes of informed consent.

### 2.3 Protecting trust in medicine

It is worth considering one additional moral purpose that informed consent plausibly plays: the protection of trust in medicine.^34^ If we think about the doctor‐patient relationship, we might expect that failing to secure informed consent can undermine trust in a particular clinician. As described above, receiving insufficient information could lead to unpleasant surprises or disastrous outcomes. Violations of patients’ trust—especially if widespread—might further weaken social trust in medical institutions. An erosion of trust in medicine might make it less likely that individuals will seek (and comply with) medical advice. Without trust in medicine, the effectiveness of the medical profession can be undermined.

Trust is especially significant to genomics, a field where public trust is already less than robust; a recent survey categorizing 43% of participants as having ‘low trust’ when it comes to the clinical use of genomic technologies.^35^ Genomic sequencing can reveal considerable amounts of personal information, with potential implications for family members. Instances of insurance and employment discrimination due to GS, and well as more general data breaches, threaten to undermine the public's acceptance of genetic technologies.

In relation to GS, an erosion of trust might make it less likely that patients follow up investigations in relation to unsolicited findings, or it might dissuade patients from disclosing relevant results to family members that are at risk of developing a genetic condition. What matters here is how effectively different informed consent models will prevent patients from experiencing the kinds of unpleasant surprises that might threaten societal trust in genomic medicine.

## 3 THE PROMICE FRAMEWORK

These accounts of the values of informed consent are not mutually exclusive; it might be important to secure informed consent for some combination of the above reasons. Indeed, we think each of the above accounts capture *something* morally important about informed consent requirements. It seems independently morally important that we respect patients’ autonomy, promote their well‐being, promote their autonomy, and preserve social trust in medicine. For those who hold this composite view, the next step will be to determine what set of informed consent requirements strikes the best balance between these four distinct moral goals.

The upshot of the above discussion is that there are multiple reasons why informed consent requirements are morally important. Critically, this also means that the underlying goals of informed consent requirements can conflict. For example, well‐being might sometimes be promoted most effectively by directing patients toward a particular course of action, whereas autonomy might be best promoted by remaining entirely non‐directive (even if some patients therefore fail to choose the course of action that best aligns with their interests). If, on the other hand, we are motivated primarily by protecting public trust in medicine, we might place special value on minimizing any possibility of a medical scandal. However, this goal can also conflict with the other moral purposes of informed consent. Extremely extensive consent standards might put a strain on resources, thereby undermining GS's ability to promote well‐being.

Yet it is important not to exaggerate how often the various goals of informed consent will conflict. The goals of promoting of autonomy, well‐being, and trust in medicine often align. For example, each of these goals suggests that it is important to provide information that is neither jargon‐filled nor needlessly complex; if the relevant information is not understood, it will not help promote autonomy or well‐being, and nor will it protect trust in medicine. Conversely, the better that patients understand the relevant risks and benefits of the options they face (with respect to their values and interests), the more fully the informed consent process will realize its various goals. When the goals of informed consent align (as they do in the case of keeping relevant information accessible), there is a strong case for adopting measures that promote every relevant goal.

These considerations underlie a new moral framework for assessing informed consent practices, which we have named the PROMICE framework (PROmoting Morally Important Consent Ends: PROMICE). The defining feature of the PROMICE framework is that it is pluralistic. Promotion of autonomy, promotion of well‐being, and protection of trust in medicine are all legitimate goals, none of which obviously trumps the others. It can be appropriate to make trade‐offs between these different goals and try to strike a balance between them. For example, it might be worth sacrificing some small degree of autonomy if this will greatly increase well‐being (or vice versa). On the other hand, when a particular approach or model satisfies all the various goals of informed consent, this is a good indication that it is worth pursuing.

Another benefit of the PROMICE framework is that it refocuses ethical inquiry away from the question of how *fully* informed a patient or research participant is and places the focus more aptly on whether their consent is ‘appropriately’ informed. By ‘appropriately’, we mean with respect to informed consent's underlying goals. This, we think, is the correct moral yardstick. Not all information is equal, and some kinds of information will be more useful to patients—and more salient to the goals of informed consent—than others.

As discussed above, advances in genomics pose new difficulties for informed consent, in that traditional benchmarks for informed consent might be unattainable. The standards that have guided informed consent practices for traditional genetic technologies are not feasible for genomic sequencing technologies, since the range of potential results can be too broad and too unpredictable to fully explain ahead of time. Even if ‘fully’ informed consent would be ideal, the unique features of GS require us to adopt some different standard. This is where the PROMICE framework proves especially useful. By identifying the moral goals of informed consent, it provides a structure for assessing the ethical appropriateness of various models for obtaining informed consent in GS.

To illustrate the value of the PROMICE framework, we will examine two controversial issues relating to obtaining informed consent in genomics and demonstrate how it can be usefully applied.

### 3.1 Informed consent processes for genomic sequencing technologies

Although GS has been incredibly useful in increasing the diagnostic yield for patients—it results in identification of the genetic cause in up to 57.5% of patients, depending on the condition and selection criteria used^36^ —large proportions of patients remain undiagnosed. Variants of uncertain significance are commonly identified and how these variants are reported to genetic health professionals (and the subsequent disclosure of these variants to patients) varies considerably between genetics services.^37^ Although much less frequently identified, unsolicited findings are also a potential outcome of diagnostic GS, particularly when using more ‘open’ analyses, rather than condition‐specific (virtual) panels.^38^ In addition, some laboratories also offer to search for variants in disease‐causing genes that are beyond the scope of the test (which are known as secondary findings).^39^ Systematic analyses of consent forms in use for diagnostic GS have identified striking variation in the degree to which these aspects are described on the forms, leading some authors to question how ‘informed’ patients’ consent might be when some of these forms are utilized.^40^ As such, it is reasonable to consider the type of consent model that might be suited to improve this process.

We will begin by looking at a model of informed consent that has been proposed for use in several applications of genomic sequencing technologies—the tiered, layered approach.^41^ We are not necessarily advocating for this approach as the best model for obtaining informed consent. Rather, we are using this as an example of how the PROMICE framework can be used in the ethical evaluation of different models of informed consent. The PROMICE framework could equally be applied to various forms of ‘generic consent’ to genetic screening,^42^ new approaches to genetic counselling,^43^ and the development of rubrics to determine how in‐depth pre‐test discussions should be and whether a genetics clinician should be involved.^44^ Considerations beyond the choice of informed consent model—such as the kinds of language used^45^ and the potential use of decision aids such as interactive webpages^46^ —may also be relevant. In each case, the PROMICE framework would help us assess how well such approaches promote informed consent's underlying moral purposes.

Although there have been several iterations, the combined tiered, layered approach was initially suggested by Bunnik and colleagues as a model for obtaining consent in the context of personal genomic (or ‘direct‐to‐consumer’) testing.^47^ In Bunnik et al.'s proposal, an ethical consent process would be (a) tiered, meaning that patients (or, in their paper, ‘consumers’) would have the option to choose different categories of results that they want to receive from testing, and (b) layered, in that there would be a minimal set of information that all patients receive, while additional information is provided to patients who wish to learn more. In their view, these components would render the necessary information accessible and understandable, while also ensuring that information considered crucial for informed decision‐making would be prioritized over that which is less important, or of mere interest.

In the context of obtaining informed consent for diagnostic genomic sequencing, patients could be presented with tiered options of the results they can receive from the test. One form this might take is outlined in Table 1.

**Table 1:** Example tiers that could be used when obtaining informed consent for diagnostic genomic sequencing

Tier 1	Results considered to be the cause of the condition under investigation
Tier 2	Variants of uncertain significance (i.e. cannot be confirmed or ruled out as the cause)
Tier 3	Unsolicited findings that are actionable (i.e. treatable, monitorable, preventable, e.g. hereditary breast and ovarian cancer)
Tier 4	Unsolicited findings that are non‐actionable (e.g. Huntington disease, dementia)
Tier 5	Information with reproductive implications (e.g. carrier status for cystic fibrosis)

Patients could opt to receive results from some tiers (e.g. unsolicited findings that are actionable) and not others (e.g., unsolicited findings that are non‐actionable).

During the consent process, patients would be presented with one set (or ‘layer’) of information that covers what is considered to be the most critical information (such as the scope, benefits and risks of the test, information about privacy and confidentiality, management of unsolicited findings, options for future use of the sample, etc.).^48^ An initial ‘layer’ of information would also be provided on each tier of potential results. Additional layers of information could be provided for patients interested in knowing more.

The following discussion focuses on the tiered and layered nature of Bunnik and colleagues’ model. However, it is worth noting that Bunnik and colleagues also intend for this model to be staged; the informed consent process is understood to take place over time, with different kinds of consent given at specific stages (e.g. before testing, before receiving results, and before receiving updates to test results). Here, too, the aim is to provide the opportunity for greater learning and understanding.

### 3.2 Autonomy

Understanding this model, we can then use the PROMICE framework to assess it. First (and most straightforwardly), the tiered and layered approach does not violate respect for autonomy in that there is no reason to think this model is more prone to deception or coercion than any other.

Second, the tiered and layered model could promote autonomy by allowing patients greater scope to decide what tier(s) of results they might wish to receive (e.g. actionable vs. non‐actionable), as opposed to allowing them only to opt in or out of a single set of findings. At the same time, the various ‘layers’ of information are designed to be maximally *useful* to patients’ deliberations regarding whether to undergo genomic testing. Notably, one of the goals of layering information is to minimize two contrasting threats to autonomous decision‐making. The first is the threat of insufficient information. If patients are to effectively decide which choice would satisfy their own values, they need sufficient information about each category of results they might decide to receive. The second is the threat of information overload, which would undermine autonomy from the other direction: by hindering effective deliberation about which option would express one's autonomy. Insofar as the tiered and layered model charts a successful path between these risks, it will help promote patient autonomy.

### 3.3 Well‐being

By seeking to avoid both insufficient information and information overload, the tiered and layered consent model also has the potential to promote well‐being. This is for two reasons.

First, by enabling patients to select which tiers of results they might like to receive, the model provides patients with greater scope to find and select an option that aligns with their interests, as they understand them. Second, by ‘layering’ the available information, the model aims to provide patients with as much information as would help them reach a decision that they feel aligns with their values (but no more information than is necessary to achieve this goal). Admittedly, this might result in some patients receiving less thorough information than if all patients were presented with extensive details. However, as we saw above, the goal of layering information is to aid decision‐making by protecting against information overload. This feature of the layered model is relevant not only to autonomy, but also to well‐being. The more fully patients understand the possible consequences of the options available to them, the more likely it is that their decision will in fact promote their well‐being.

### 3.4 Trust

These same features of the tiered and layered model could help protect trust in medicine. By ensuring that the critical information required for informed decision‐making is covered in a comprehensible way, and not lost within exhaustive details that are less important (but potentially interesting to some), the tiered and layered model should help prevent the kind of situations that might undermine trust in medicine. Specifically, the model should minimize the possibility that information will be forgotten, misremembered, or misunderstood, with the attendant risks for these patients' trust in medicine and/or the production of scandals that undermine public confidence in genomic medicine more broadly.

### 3.5 PROMICE in action

The PROMICE framework usefully illustrates how ‘less can be more’ when it comes to obtaining informed consent from some patients, as well as the importance of focusing on *appropriately* (rather than *fully*) informed consent in complex areas such as genomics. By appropriately, we mean that the four morally important goals we have described are addressed; consent is *appropriately* informed when it achieves the underlying goals that informed consent is meant to achieve. As we have outlined, it is not necessary for patients to understand absolutely every aspect related to genomic sequencing in order to promote autonomy and well‐being, nor to prevent trust‐undermining situations. Rather than merely focusing on information provision, the focus should be on helping patients make decisions that align with their values.

Our application of the PROMICE framework to the tiered and layered consent model suggests that it is a promising approach to achieving appropriately informed consent. However, further research needs to be undertaken to explore which information is most relevant to patients so that consent processes can be designed to meet their needs.

## 4 RAPID GENOMIC TESTING IN THE NICU

One setting where considerations of informed consent are particularly challenging is the use of GS in critically ill children. Using GS to diagnose genetic disorders in the NICU has already shown significant promise with large proportions of patients receiving diagnoses over several studies, many of which lead to important changes to patient management.^49^ Unfortunately, the standard turnaround time for GS is 3–6 months, which can limit the clinical utility in acute cases.^50^ However, the recent implementation of ‘rapid’ GS (RGS) in the research setting, with a turnaround time of 2–21 days, is increasing the potential for use of GS in the NICU setting. By providing a faster diagnosis and therefore the potential for expedited intervention, rapid GS can amplify the benefits of standard sequencing and, ultimately, lead to better patient care.

However, several aspects of the NICU setting pose unique challenges for the informed consent process.^51^ In conjunction with the complexity of the information being provided, the urgency of the NICU adds a significant time pressure to parents’ decision‐making about whether or not to agree to their baby participating in research‐based rapid GS.^52^ On top of this, the NICU is a particularly stressful environment for parents as their child's life is often hanging in the balance; accordingly, parents may feel pressured to agree to any test that may increase the chances of their child's survival. This may result in decisions being made without adequate reflection on the full implications of GS,^53^ such as the potential outcomes of the test or how comfortable they are with any data sharing requirements associated with the study. Finally, the blurred boundary between clinical care and research when these studies are being conducted within an acute setting may result in confusion for parents regarding which aspects are research versus which are part of the standard of care.

The PROMICE framework can help identify key ethical considerations for informed consent in this setting. Specifically, the PROMICE framework tells us to balance three considerations when assessing informed consent processes.

### 4.1 Autonomy

One of the reasons RGS is such a complex procedure is that it usually involves three different tests. In order to analyse an infant's genome efficiently, you need to compare it with the genome of their parents. This means consent is often needed to perform genomic sequencing on three different samples—referred to as trio sequencing. Questions of the promotion of autonomy apply differently to each of these samples. For each parent's sample, we should promote autonomy in decision‐making in the standard ways: by ensuring parents have accurate and up‐to‐date information about the test to allow them to make decisions according to their own personal values. However, for the child's sample, the decision about whether sequencing should occur should not be made according to the parents’ personal values. This is because this decision is actually a case of ‘proxy’ decision‐making; the children's parents are being asked to consent to sequencing of their child's sample, on behalf on their child.

It is not obvious how we can promote the autonomy of an infant, who themselves are not fully autonomous agents. However, as death takes away all options (and prevents autonomy from being exercised or developing further), the promotion of autonomy favours decisions that maximize a child's chances of surviving to adulthood.

### 4.2 Well‐being

One of the unique features of rapid GS is its potential to result in large net increases to well‐being.^54^ In some cases, RGS will identify a potentially fatal problem with metabolism or an immune deficiency that can be quickly treated. In these cases, a child who would have died in infancy now has a normal life expectancy as a direct result of a diagnosis made possible through rapid GS. Only very few interventions have such a large potential influence on individual well‐being. For a much larger group of children, rapid GS promotes well‐being in significant but less pronounced ways, for example by reducing the need for painful and invasive investigations. It may reveal a poor prognosis that will result in a decision to limit life‐sustaining treatment and the death of the infant.

Where having a medical procedure is clearly in someone's best interest, we are sometimes justified in applying different informed consent procedures, particularly if failing to do so could cause significant harm. We already accept this principle in relation to emergency situations where people are dying and unconscious, and weighty medical decisions (such as whether to perform a blood transfusion) must be made under severe time constraints. In these cases we may be entitled to presume consent, because on most (but not all) views of what a good life is, having a life‐saving blood transfusion is in your best interest. Conversely, delaying urgently needed treatment in the hopes of securing informed consent would (in most such cases) be contrary to the patient's best interests. The same is true in the context of RGS of critically ill children; if time pressures make ‘fully’ informed consent unfeasible in the moment, it may be acceptable to temporarily relax informed consent standards (although afterwards parents or patients should still be fully informed, and their consent remains important). One of the key goals of informed consent is to promote well‐being; however, under some circumstances, time pressures mean that an involved informed consent process can impede this goal by delaying urgent treatment. If this goal is best met by adopting a more flexible approach to consent, then (at least from the standpoint of well‐being) we ought to do so.

### 4.3 Trust

Whereas the previous considerations favour a fast, directive consent procedure, considerations of trust in medicine point in the opposite direction. As described earlier, some segments of society are still distrustful of genomic technologies. Fast, directive approaches to consent may not allow people to critically reflect on all possible implications of their decisions. Significantly, RGS can have some unexpected consequences. For example, there is reason to believe that GS in the newborn period can affect parent‐child bonding and interfere with family dynamics.^55^ This could lead to regret, with parents’ negative experiences (and any associated scandals) potentially undermining societal trust in genomic medicine more generally. This consideration favours ensuring, where possible, that people understand the broader implications of RGS.

### 4.4 PROMICE in practice

The PROMICE framework identifies the crucial elements to consider when seeking informed consent for rapid GS in critically ill children. First, the potential for RGS to be life‐saving means it is in the best interest for critically ill children. Second, the fact that RGS involves an element of proxy decision‐making complicates how we promote autonomy in this case, and again focuses our attention on the fact that RGS can be autonomy‐promoting for the child. Third, the fact that many people have low trust in genomic technologies, coupled with the implications sequencing can have for one's personal life and one's relatives, highlights the need for a consent pathway that allows efficient access to RGS but can also accommodate those with concerns regarding RGS to carefully weigh their decision. Information provided during the informed consent process should include issues related to trust of genomic sequencing, such as possible uses of data and insurance implications.

### 4.5 Limitations of the PROMICE framework

We have attempted to outline the key moral purposes of informed consent requirements and sketched their application to live controversies in GS. We have not, however, taken a stance on how important it is to secure informed consent, all things considered. There is a rich literature in sociology and bioethics that critiques the importance often ascribed to informed consent.^56^

A common refrain in such literature is that focusing myopically on informed consent might lead us to miss important moral issues to which informed consent is not a solution. For example, it might lead us to neglect power dynamics in the relationship between doctors and patients or research subjects, or considerations about what kinds of research or treatment are too risky to be allowed even if they are freely consented‐to. The value of the PROMICE framework lies in helping achieve the moral goals of informed consent; however, it is worth acknowledging that these goals are not the only ones that matter.

## 5 CONCLUSIONS

Respect for autonomy is an important moral principle and, in line with this principle, it is important that consent to GS is free of coercion and deception. But respect for autonomy alone does not provide a full justification for informed consent to GS. As we have argued, there are other morally important goals that are promoted by the informed consent process. These include the promotion of autonomy, the promotion of well‐being, and the preservation of trust in medicine.

There are important distinctions between each of these goals and, in some cases, they might conflict. For example, if we understand the moral purpose of informed consent mostly in terms of promoting well‐being, we might be comfortable with a greater degree of paternalism than if we understand the moral purpose of informed consent mostly in terms of promoting autonomy. Importantly, however, there is a crucial area of overlap between these goals. Each account of informed consent's value would presumably recommend providing patients with the kind of information they would find most helpful to their decision‐making (which would, in turn, plausibly promote well‐being, autonomy, and trust in medicine). These goals are best achieved not by providing some maximal amount of (potentially highly complex and technical) information, but rather by seeking the best possible balance between comprehensiveness on the one hand, and comprehensibility and clarity on the other. As we demonstrated with the examples, the best way to do so is context‐dependent and needs be assessed for each testing situation.

Of course, we are still faced with the challenge of determining what constitutes relevant information for patients/participants in the face of genomic medicine. This is likely to be based on a number of factors, including their clinical situation, level and area of education, personal values, and the time they are willing to devote to the consent process. It would be useful to conduct further research to explore patients’ experiences with, and preferences for, information provided during the consent process.

‘Fully’ informed consent might be unattainable because genomics is too complex for a member of the general public to understand. However, we have also argued that ‘fully’ informed consent is the wrong standard to use. We should instead ask how we can best achieve the underlying moral purposes of informed consent in the context of GS. As we have shown, informed consent can promote a range of morally important goals. If the information provided to patients allows for promotion of these goals, then the patient can give sufficiently, and more importantly, *appropriately* informed consent.

## CONFLICTS OF INTEREST

The authors declare no conflicts of interest.

## Footnotes

1

Clarke, A. J. (2014). Managing the ethical challenges of next‐generation sequencing in genomic medicine. British Medical Bulletin, 111, 17–30; Koplin, J. J., Savulescu, J., & Vears, D. F. (2020). Why genomics researchers are sometimes morally required to hunt for secondary findings. *BMC Medical Ethics, 21*, 1–11.3200522510.1186/s12910-020-0449-8PMC6995186

2

Vears, D. F. , Borry, P. , Savulescu, J. , & Koplin, J. J. (2020). Old challenges or new issues? Genetic health professionals' experiences obtaining informed consent in diagnostic genomic sequencing. AJOB Empirical Bioethics, 12, 12–23.3301726510.1080/23294515.2020.1823906PMC8120994

3

Eyal, N. (2011). Informed consent. In E. N. Zalta (Ed.), *Stanford encyclopedia of philosophy*. Stanford University

4

Dondorp, W. J. , & De Wert, G. M. (2013). The ‘thousand‐dollar genome’: An ethical exploration. European Journal of Human Genetics, 21, S6–S26; Bradbury, A. R., Patrick‐Miller, L., & Domchek, S. (2016). Multiplex genetic testing: Reconsidering utility and informed consent in the era of next‐generation sequencing. *Genetics in Medicine, 17*, 97–98; Burke, K., & Clarke, A. (2016). The challenge of consent in clinical genome‐wide testing. *Archives of Disease in Childhood, 101*, 1048–1052; Burke, W., Beskow, L. M., Trinidad, S. B., Fullerton, S. M., & Brelsford, K. (2018). Informed consent in translational genomics: Insufficient without trustworthy governance. *The Journal of Law, Medicine & Ethics, 46*, 79–86. These problems were anticipated by the Secretary's Advisory Committee on Genetic Testing before the human genome project had even concluded. Barbara Koenig describes how ‘[e]ven then, we recognized that the ideal of full disclosure of all risks and benefits of a particular genetic test, ideally by a trained genetic counselor, would collapse once the volume of genomic data increased. If it took an hour to counsel a patient about one condition, what would happen if panels of test could simultaneously offer multiple findings?’ Koenig, B. A. (2014). Have we asked too much of consent? *Hastings Center Report, 44*, 33–34.23677179

5
Vears et al., op. cit. note 2.

6

Gyngell, C. , Newson, A. J. , Wilkinson, D. , Stark, Z. , & Savulescu, J. (2019). Rapid challenges: Ethics and genomic neonatal intensive care. Pediatrics, 143, S14–S21.3060026610.1542/peds.2018-1099DPMC6379057

7

Millum, J. , & Bromwich, D. (2021). Informed consent: What must be disclosed and what must be understood? The American Journal of Bioethics, 21, 46–58; Beauchamp, T. L. (2011). Informed consent: Its history, meaning, and present challenges. *Cambridge Quarterly of Healthcare Ethics, 20*, 515–523; Grady, C. (2015). Enduring and emerging challenges of informed consent. *New England Journal of Medicine, 372*, 855–862; Henderson, G. E. (2011). Is informed consent broken? *American Journal of Medical Science, 342*, 267–272; O'Neill, O. (2003). Some limits of informed consent. *Journal of Medical Ethics, 29*, 4–7.

8
Vears et al., op. cit. note 2.

9

Hurd, H. M. (2005). Blaming the victim: A response to the proposal that criminal law recognize a general defense of contributory responsibility. Buffalo Criminal Law Review, 8, 503–522.

10

Faden, R. R. , & Beauchamp, T. L. (1986). A history and theory of informed consent. Oxford University Press.

11
Eyal, op. cit. note 3.

12

Pugh, J. (2020). Informed consent, autonomy, and beliefs. InAutonomy, rationality, and contemporary bioethics (pp. 149–182). Oxford University Press.32396289

13

Beauchamp, T. L. , & Childress, J. F. (2001). Principles of biomedical ethics. Oxford University Press.

14

Sullivan, M. (2016) The patient as agent of health and health care: Autonomy in patient‐centered care for chronic conditions. Oxford University Press.

15

Taylor, J. S. (2009). *Practical autonomy and bioethics*. Routledge.

16

O'Neill, O. (2002). Autonomy and trust in bioethics. Cambridge University Press; Ibid; Manson, N. C., & O'Neill, O. (2007). *Rethinking informed consent in bioethics*. Cambridge University Press.

17
See, e.g. Beauchamp & Childress, op. cit. note 13; Faden, R. R. , Beauchamp, T. L. , & King, N. M. P. (1986). A history and theory of informed consent. Oxford University Press; Dworkin, G. (1988). *The theory and practice of autonomy*. Cambridge University Press; Young, R. (1998). Informed consent and patient autonomy. In H. Kuhse & P. Singer (Eds.), *A companion to bioethics* (pp. 441–451). Wiley‐Blackwell.

18

Christman, J. (2008). Autonomy in moral and political philosophy. In E. N. Zalta (Ed.), Stanford encyclopedia of philosophy. Stanford University. James Taylor offers the following general definition of respect for autonomy: ‘for A to respect the autonomy of B she must intentionally allow him to make his own decisions in light of his own beliefs and values, and refrain from subjecting him to coercion, duress, manipulation, or deception’. Taylor, J. S. (2009). *Practical autonomy and bioethics*. Routledge, p. 136.

19
Taylor, op. cit. note 15.

20
Informed consent processes might nonetheless provide some useful assurance that patients (and/or research subjects) have not been deceived or coerced. See: O'Neill, op. cit. note 7.

21
We treat respect for autonomy and promotion of autonomy as independent goals. However, promotion of autonomy could arguably be described as a component of respect for autonomy (since, if we respect autonomy, we also ought to promote it). Even if this is correct, however, the two goals we have been discussing remain somewhat distinct, in that what we have called ‘respect for autonomy’ is a negative duty and what we have called ‘promotion of autonomy’ is a positive one.

22
Beauchamp & Childress, op. cit. note 13, p. 58.

23

Savulescu, J. , & Momeyer, R. W. (1997). Should informed consent be based on rational beliefs? Journal of Medical Ethics, 23, 282–288.935834710.1136/jme.23.5.282PMC1377366

24

Dive, L. , & Newson, A. J. (2018). Reconceptualizing autonomy for bioethics. Kennedy Institute of Ethics Journal, 28, 171–203.3010059910.1353/ken.2018.0013

25

Berlin, I. , Hardy, H. , & Harris, I. (2002). Liberty: Incorporating four essays on liberty. Oxford University Press.

26

Carter, I. (2003). Positive and negative liberty. In E. N. Zalta (Ed.),Stanford encyclopedia of philosophy. Stanford University.

27
Taylor, op. cit. note 15.

28
It could be argued that autonomy is one facet of well‐being—in which case the goal of promoting autonomy can be subsumed within the goal of promoting well‐being. We think our framework can readily accommodate this view—one would merely need to distinguish between promoting autonomy and promoting other facets of well‐being.

29
Beauchamp & Childress, op. cit. note 13.

30

Conly, S. (2013). Against autonomy: Justifying coercive paternalism. Cambridge University Press; Levy, N. (2014). Forced to be free? Increasing patient autonomy by constraining it. *Journal of Medical Ethics, 40*, 293–300; Caplan, A. L. (2014). Why autonomy needs help. *Journal of Medical Ethics, 40*, 301–302.

31
Vears et al., op. cit. note 2.

32
We are here treating directiveness as contrary to informed consent. It is worth noting that some argue that cleaving too closely to nondirectiveness in genetic counselling can itself undermine autonomy, since the mere provision of information is less useful for clients’ decision‐making than meaningful dialogue. See: Hodgson, J., & Spriggs, M. (2005). A practical account of autonomy: Why genetic counseling is especially well suited to the facilitation of informed autonomous decision making.Journal of Genetic Counseling,14, 89–97.10.1007/s10897-005-4067-x15959640

33
Note that we are not saying that the value of informed consent is wholly reducible to the value of patient well‐being. To the contrary, the PROMICE framework ultimately adopts a pluralistic account of informed consent's value(s). These additional goals mean that it is not, as a rule, appropriate to abandon informed consent wherever we can more effectively promote autonomy by violating it.

34

Manson & O'Neill, op. cit. note 16; O'Neill, op. cit. note 16 ; Tännsjö, T. (2014). Utilitarianism and informed consent. Journal of Medical Ethics, 40, 445–445. The protection of trust cannot be considered the *only* moral purpose of informed consent. Among other problems, this view would entail that it is acceptable to violate informed consent requirements whenever this violation would go undetected. See: Eyal, N. (2014). Using informed consent to save trust. *Journal of Medical Ethics, 40*, 437–444.23303176

35

Milne, R. , Morley, K. I. , Howard, H. , Niemiec, E. , Nicol, D. , Critchley, C. , Prainsack, B. , Vears, D. F. , Smith, J. , & Steed, C. (2019). Trust in genomic data sharing among members of the general public in the UK, USA, Canada and Australia. Human Genetics, 138, 1237–1246.3153174010.1007/s00439-019-02062-0PMC6874520

36

Yang, Y. , Muzny, D. M. , Xia, F. , Niu, Z. , Person, R. , Ding, Y. , Ward, P. , Braxton, A. , Wang, M. , Buhay, C. , Veeraraghavan, N. , Hawes, A. , Chiang, T. , Leduc, M. , Beuten, J. , Zhang, J. , He, W. , Scull, J. , Willis, A. , … Eng, C. M. (2014). Molecular findings among patients referred for clinical whole‐exome sequencing. 
JAMA
, 312, 1870–1879; Yang, Y., Muzny, D. M., Reid, J. J. G., Bainbridge, M. N., Willis, A., Ward, P. A., Braxton, A., Beuten, J., Xia, F., Niu, Z., Hardison, M., Person, R., Bekheirnia, M. R., Leduc, M. S., Kirby, A., Pham, P., Scull, J., Wang, M., Ding, Y., … Eng, C. M. (2013). Clinical whole‐exome sequencing for the diagnosis of Mendelian disorders. *New England Journal of Medicine, 369*, 1502–1511; Lee, H., Deignan, J. L., Dorrani, N., Strom, S. P., Kantarci, S., Quintero‐Rivera, F., Das, K., Toy, T., Harry, B., Yourshaw, M., Fox, M., Fogel, B. L., Martinez‐Agosto, J. A., Wong, D. A., Chang, V. Y., Shieh, P. B., Palmer, C. G., Dipple, K. M., Grody, W. W., … Nelson, S. F. (2014). Clinical exome sequencing for genetic identification of rare Mendelian disorders. *JAMA, 312*, 1880–1887; Soden, S. E., Saunders, C. J., Willig, L. K., Farrow, E. G., Smith, L. D., Petrikin, J. E., LePichon, J. B., Miller, N. A., Thiffault, I., Dinwiddie, D. L., Twist, G., Noll, A., Heese, B. A., Zellmer, L., Atherton, A. M., Abdelmoity, A. T., Safina, N., Nyp, S. S., Zuccarelli, B., … Kingsmore, S. F. (2014). Effectiveness of exome and genome sequencing guided by acuity of illness for diagnosis of neurodevelopmental disorders. *Science Translational Medicine, 6*, 265ra168; Daoud, H., Luco, S. M., Li, R., Bareke, E., Beaulieu, C., Jarinova, O., Carson, N., Nikkel, S. M., Graham, G. E., Richer, J., Armour, C., Bulman, D. E., Chakraborty, P., Geraghty, M., Lines, M. A., Lacaze‐Masmonteil, T., Majewski, J., Boycott, K. M., & Dyment, D. A. (2016). Next‐generation sequencing for diagnosis of rare diseases in the neonatal intensive care unit. *CMAJ: Canadian Medical Association Journal = journal de l'Association medicale canadienne, 188*, E254–E260; Stark, Z., Tan, T. Y., Chong, B., Brett, G. R., Yap, P., Walsh, M., Yeung, A., Peters, H., Mordaunt, D., Cowie, S., Amor, D. J., Savarirayan, R., McGillivray, G., Downie, L., Ekert, P. G., Theda, C., James, P. A., Yaplito‐Lee, J., Ryan, M. M., … White, S. M. (2016). A prospective evaluation of whole‐exome sequencing as a first‐tier molecular test in infants with suspected monogenic disorders. *Genetics in Medicine: Official Journal of the American College of Medical Genetics, 18*, 1090–1096.10.1038/gim.2016.126938784

37

Vears, D. F. , Senecal, K. , & Borry, P. (2017). Reporting practices for variants of uncertain significance from next generation sequencing technologies. European Journal of Medical Genetics, 60, 553–558.2877484810.1016/j.ejmg.2017.07.016

38

Vears, D. F. , Sénécal, K. , Clarke, A. J. , Jackson, L. , Laberge, A. M. , Lovrecic, L. , Piton, A. , Van Gassen, K. L. I. , Yntema, H. G. , Knoppers, B. M. , & Borry, P. (2018). Points to consider for laboratories reporting results from diagnostic genomic sequencing. European Journal of Human Genetics, 26, 36–43.2918417110.1038/s41431-017-0043-9PMC5839050

39

Kalia, S. S. , Adelman, K. , Bale, S. J. , Chung, W. K. , Eng, C. , Evans, J. P. , Herman, G. E. , Hufnagel, S. B. , Klein, T. E. , Korf, B. R. , McKelvey, K. D. , Ormond, K. E. , Richards, C. S. , Vlangos, C. N. , Watson, M. , Martin, C. L. , & Miller, D. T. (2017). Recommendations for reporting of secondary findings in clinical exome and genome sequencing, 2016 update (ACMG SF v2.0): A policy statement of the American College of Medical Genetics and Genomics. Genetics in Medicine, 19, 249–255.2785436010.1038/gim.2016.190

40

Vears, D. F. , Niemiec, E. , Howard, H. C. , & Borry, P. (2018). How do consent forms for diagnostic high‐throughput sequencing address unsolicited and secondary findings? A content analysis. Clinical Genetics, 94, 321–329; Vears, D. F., Niemiec, E., Howard, H. C., & Borry, P. (2018). Analysis of VUS reporting, variant reinterpretation and recontact policies in clinical genomic sequencing consent forms. *European Journal of Human Genetics, 26*, 1743–175129888485

41

Bunnik, E. M. , Janssens, A. C. J. , & Schermer, M. H. (2013). A tiered‐layered‐staged model for informed consent in personal genome testing. European Journal of Human Genetics, 21, 596–607; Bradbury, A. R., Patrick‐Miller, L., Long, J., Powers, J., Stopfer, J., Forman, A., Rybak, C., Mattie, K., Brandt, A., & Chambers, R. (2015). Development of a tiered and binned genetic counseling model for informed consent in the era of multiplex testing for cancer susceptibility. *Genetics in Medicine, 17*, 485‐–492.23169494

42

Elias, S. , & Annas, G. J. (1994). Generic consent for genetic screening. New England Journal of Medicine, 330, 1611–1613.817725310.1056/NEJM199406023302213

43

Ormond, K. E. (2013). From genetic counseling to “genomic counseling”. Molecular Genetics & Genomic Medicine, 1, 189–193.2449861510.1002/mgg3.45PMC3865587

44

Ormond, K. E. , Hallquist, M. L. , Buchanan, A. H. , Dondanville, D. , Cho, M. K. , Smith, M. , Roche, M. , Brothers, K. B. , Coughlin, C. R. , & Hercher, L. (2019). Developing a conceptual, reproducible, rubric‐based approach to consent and result disclosure for genetic testing by clinicians with minimal genetics background. Genetics in Medicine, 21, 727–735.2997698810.1038/s41436-018-0093-6PMC6320736

45

Greenwood, J. , & Crowden, A. (2021). Thinking about the idea of consent in data science genomics: How ‘informed’ is it? Nursing Philosophy, 22, e12347.3397947410.1111/nup.12347

46

Rego, S. , Grove, M. E. , Cho, M. K. , & Ormond, K. E. (2020). Informed consent in the genomics era. Cold Spring Harbor Perspectives in Medicine, 10, a036582.3157038210.1101/cshperspect.a036582PMC7397836

47
Bunnik et al., op. cit. note 41.

48
For a full list of suggested minimal information to be included in consent forms for genomic sequencing, see: Ayuso, C., Millán, J. M., Mancheno, M., & Dal‐Ré, R. (2013). Informed consent for whole‐genome sequencing studies in the clinical setting. Proposed recommendations on essential content and process. *European Journal of Human Genetics*, *21*(10), 1054–1059.10.1038/ejhg.2012.297PMC377833623321621

49

Farnaes, L. , Hildreth, A. , Sweeney, N. M. , Clark, M. M. , Chowdhury, S. , Nahas, S. , Cakici, J. A. , Benson, W. , Kaplan, R. H. , Kronick, R. , Bainbridge, M. N. , Friedman, J. , Gold, J. J. , Ding, Y. , Veeraraghavan, N. , Dimmock, D. , & Kingsmore, S. F. (2018). Rapid whole‐genome sequencing decreases infant morbidity and cost of hospitalization. NPJ Genomic Medicine, 3, 10; Daoud, H., Luco, S. M., Li, R., Bareke, E., Beaulieu, C., Jarinova, O., Carson, N., Nikkel, S. M., Graham, G. E., Richer, J., Armour, C., Bulman, D. E., Chakraborty, P., Geraghty, M., Lines, M. A., Lacaze‐Masmonteil, T., Majewski, J., Boycott, K. M., & Dyment, D. A. (2016). Next‐generation sequencing for diagnosis of rare diseases in the neonatal intensive care unit. *CMAJ: Canadian Medical Association Journal = journal de l'Association medicale canadienne, 188*, E254–E260; Stark, Z., Lunke, S., Brett, G. R., Tan, N. B., Stapleton, R., Kumble, S., Yeung, A., Phelan, D. G., Chong, B., Fanjul‐Fernandez, M., Marum, J. E., Hunter, M., Jarmolowicz, A., Prawer, Y., Riseley, J. R., Regan, M., Elliott, J., Martyn, M., Best, S., … Melbourne, A. (2018). Genomics health. Meeting the challenges of implementing rapid genomic testing in acute pediatric care. *Genetics in Medicine, 20*, 1554–1563; van Diemen, C. C., Kerstjens‐Frederikse, W. S., Bergman, K. A., de Koning, T. J., Sikkema‐Raddatz, B., van der Velde, J. K., Abbott, K. M., Herkert, J. C., Lohner, K., Rump, P., Meems‐Veldhuis, M. T., Neerincx, P. B. T., Jongbloed, J. D. H., van Ravenswaaij‐Arts, C. M., Swertz, M. A., Sinke, R. J., van Langen, I. M., & Wijmenga, C. (2017). Rapid targeted genomics in critically ill newborns. *Pediatrics, 140*(4), e20162854; Willig, L. K., Petrikin, J. E., Smith, L. D., Saunders, C. J., Thiffault, I., Miller, N. A., Soden, S. E., Cakici, J. A., Herd, S. M., Twist, G., Noll, A., Creed, M., Alba, P. M., Carpenter, S. L., Clements, M. A., Fischer, R. T., Hays, J. A., Kilbride, H., McDonough, R. J., … Kingsmore, S. F. (2015). Whole‐genome sequencing for identification of Mendelian disorders in critically ill infants: A retrospective analysis of diagnostic and clinical findings. *Lancet Respiratory Medicine, 3*, 377–387.29644095

50

Stark, Z., et al., op. cit. note 36.

51

RGS in the NICU is also ethically controversial for other reasons .See: Deem, M. J. (2016). Whole‐genome sequencing and disability in the NICU: Exploring practical and ethical challenges. Pediatrics, 137, S47–S55. We are interested here, however, only in the challenges related to informed consent.2672970310.1542/peds.2015-3731IPMC9923973

52
Gyngell et al., op. cit. note 6.

53
Ibid.

54

Ibid ; Dimmock, D. P. , Clark, M. M. , Gaughran, M. , Cakici, J. A. , Caylor, S. A. , Clarke, C. , Feddock, M. , Chowdhury, S. , Salz,xs L. , & Cheung, C. (2020). An RCT of rapid genomic sequencing among seriously ill infants results in high clinical utility, changes in management, and low perceived harm. The American Journal of Human Genetics, 107, 942–952.3315700710.1016/j.ajhg.2020.10.003PMC7675004

55
Gyngell et al., op. cit. note 6.

56

See: Corrigan, O . (2003). Empty ethics: The problem with informed consent. Sociology of Health & Illness, 25, 768–792; O'Neill, op. cit. note 7.1978020510.1046/j.1467-9566.2003.00369.x

